# Glycemic Control as an Early Prognostic Marker in Advanced Pancreatic Cancer

**DOI:** 10.3389/fonc.2021.571855

**Published:** 2021-02-25

**Authors:** Ipek Alpertunga, Rabail Sadiq, Deep Pandya, Tammy Lo, Maxim Dulgher, Sarah Evans, Bridget Bennett, Nancy Rennert, Richard C. Frank

**Affiliations:** ^1^Department of Medicine, Norwalk Hospital, Nuvance Health, Norwalk, CT, United States; ^2^Rudy L. Ruggles Biomedical Research Institute, Nuvance Health, Danbury, CT, United States; ^3^Department of Medicine, Danbury Hospital, Nuvance Health, Danbury, CT, United States; ^4^Department of Nutrition, Norwalk Hospital, Nuvance Health, Norwalk, CT, United States

**Keywords:** pancreatic cancer, diabetes mellitus, hyperglycemia, prognostic marker, glycemic control

## Abstract

**Purpose:**

Impaired glucose metabolism is present in most patients with pancreatic ductal adenocarcinoma (PDAC). Whereas previous studies have focused on pre-treatment glycemic indices and prognosis in those with concomitant diabetes, the effects of glycemic control during chemotherapy treatment on prognosis, in patients with and without diabetes, have not been well characterized. We examined the relationship between early glycemic control and overall survival (OS) in a cohort of patients with advanced PDAC treated in a community setting.

**Patients and Methods:**

Seventy-three patients with advanced PDAC (38% with diabetes) receiving chemotherapy while participating in a biobanking clinical trial were included. Clinical characteristics and laboratory results during 1 year were obtained from the electronic medical record. Kaplan-Meier estimate, log-rank test and hazard ratios were computed to assess the effect of glycemic control on OS. The Cox proportional hazards regression model was applied to ascertain the significance of glycemic control with other survival variables.

**Results:**

One thousand four hundred eighteen random blood glucose (RBG) values were analyzed. In accord with previous findings, a 50% decline in the serum tumor marker CA 19-9 at any time was predictive of survival (*P*=0.0002). In univariate analysis, an elevated pre-treatment average RBG, 3-month average RBG (RBG-3) and the FOLFIRINOX regimen were associated with longer survival. Based on ROC analysis (AUC=0.82), an RBG-3 of 120 mg/dl was determined to be the optimal cutoff to predict 12-month survival. In multivariate analysis that included age, stage, BMI, performance status, presence of diabetes, and chemotherapy regimen, only RBG-3 maintained significance: an RBG-3 ≤120 mg/dl predicted for improved OS compared to >120 mg/dl (19 *vs.* 9 months; HR=0.37, *P*=0.002). In contrast, an early decline in CA 19-9 could not predict OS.

**Conclusion:**

Lower glucose levels during the first 3 months of treatment for advanced PDAC predict for improved OS in patients both with and without diabetes. These results suggest that RBG-3 may be a novel prognostic biomarker worthy of confirmation in a larger patient cohort and that studies exploring a possible cause and effect of this novel survival-linked relationship are warranted.

## Introduction

Pancreatic ductal adenocarcinoma (PDAC) and diabetes mellitus (DM) share a complex relationship and are both pathogenically and temporally linked. Impaired glucose tolerance is present in most patients diagnosed with PDAC and between 25%–50% have pre-existing DM, the majority classified as new-onset (1, 2). Extensive epidemiology research has shown that long-standing (LS) diabetes (>3 years duration) increases the risk of PC nearly 2-fold whereas new-onset diabetes (NOD) (variably defined as <1–3 years duration) is associated with a nearly 8-fold risk in individuals over 50 years of age (3). While LS diabetes is typically Type 2, NOD that precedes PC is classified as Type 3c (pancreatogenic) and is hypothesized to result from insulin resistance mediated by extracellular vesicles released by subclinical PDAC (4). Because of this paraneoplastic harbinger of disease, individuals with NOD are the subject of several ongoing clinical trials aimed at the early detection of PDAC (ClinicalTrials.gov ID: NCT03937453, NCT03731637).

One of the hallmarks of cancer at the cellular level is the reprogramming of energy metabolism in order to provide the necessary fuel for cell survival, proliferation and metastasis (5). During this process, glucose as the primary fuel source, is preferentially metabolized through the (anaerobic) glycolytic pathway rather than the more efficient mitochondrial oxidative phosphorylation (OXPHOS) pathway. This phenomenon is referred to as the “Warburg effect,” in honor of the German physicist Otto Warburg who pioneered its discovery (6). As it relates to pancreatic cancer, the central oncogenic driver, mutant *KRAS*, directly regulates four key steps in metabolic reprogramming: glucose import and phosphorylation, fructose-1-6-bisphosphate production and lactate export (7, 8). In addition, oncogenic *KRAS* has also recently been shown to co-opt the tumor microenvironment (TME) to sustain cancer cell glycolytic flux by directing paracrine signaling involving Type II cytokines secreted by Th2 cells in the TME (9). Furthermore, the hypoxic tumor environment that characterizes many cancers including PDAC, stimulates glycolysis through overexpression of the transcription factors HIF1α and HIF2α (5), Alterations in both glycolysis and OXPHOS have been linked to the survival, metastatic spread and chemotherapy resistance of PDAC cells (10–13).

Given the important role of glucose in malignant cell behavior, the relationship between glycemic indices, diabetes and cancer outcomes in adults has been studied across multiple tumor types, including bladder, breast, cervical, endometrial, gastroesophageal, hepatocellular, lung, ovarian and pancreatic cancers as well as glioblastoma (14–18), Most studies have analyzed the effects of pre-treatment glucose and/or HbA1c values in those with DM on prognosis and found significant correlations between poor glycemic control and adverse outcomes. In comparison, very few studies have reported on whether changes in glycemic indices during cancer treatment in those with and without DM can be used for prognostic purposes.

This latter issue is especially relevant to pancreatic cancer, in which pre-existing glucose intolerance, coupled with the presence of a primary pancreatic tumor mass and treatment with surgery, radiation and/or chemotherapy may lead to progressive β-cell loss and diminished glycemic control (19). On the other hand, approximately 50% of individuals with NOD who undergo PDAC resection experience resolution of their DM postoperatively (20). This supports the paraneoplastic hypothesis of PDAC-associated NOD and raises the specter that improved cancer control may improve glycemic control even in the advanced disease setting.

Previous studies in the setting of resectable PDAC have demonstrated that patients who have improvements in HbA1c after surgery or in average random blood glucose levels after radiation therapy had improved overall survivals (21, 22). There have not been similar analyses, however, in the setting of unresectable (Stage III) and metastatic (Stage IV) PDAC treated with effective systemic regimens, such as FOLFIRINOX (consisting of 5-fluorouracil, leucovorin, irinotecan, and oxaliplatin) and gemcitabine/nab-paclitaxel (G/N) (23, 24).

In the present study, we sought to explore the dynamic relationship between glycemic control and survival during chemotherapy treatments for individuals with advanced stage PDAC enrolled in a community hospital-based biobank study (ClinicalTrials.gov ID: NCT04406831). As this was a “real-world” cohort, we relied on serial random blood glucose (RBG) levels rather than HbA1c, which is not typically tracked by oncologists. The primary objective was to determine the relationship, if any, between glycemic control during the first 3 months of treatment and overall survival. A better understanding of this relationship could have not only prognostic implications but also therapeutic and mechanistic ones. We also sought to compare glycemic control with more standard prognostic markers, such as the tumor marker CA 19-9 (25), early in the course of treatment.

## Materials and Methods

### Patient Population

Consecutive patients treated at Norwalk, Danbury and New-Milford Hospitals in CT (part of Nuvance Health) with a diagnosis of locally advanced/unresectable or metastatic PDAC (American Joint Committee on Cancer stages III and IV, respectively) who provided informed written consent to participate in a local institutional review board approved biobank study were included. All patients underwent computed tomography (CT) imaging for staging purposes and either endoscopic ultrasound FNA/biopsy of the pancreatic primary or liver biopsy for histologic confirmation. Resectability was determined at a multi-disciplinary tumor board using standardized criteria; borderline resectable cases which proceeded to surgery after neoadjuvant therapy (chemotherapy +/- radiation) were excluded from analysis. Routine blood work typically included a CBC prior to each chemotherapy session, chemistry panel at least every 2 weeks and monthly CA 19-9 tumor marker levels. In cases of biliary obstruction requiring biliary stenting, baseline CA 19-9 values were obtained once the bilirubin normalized. Hemoglobin A1c (HbA1c) levels were obtained at the discretion of the treating physician. All patients were previously untreated and 92% received multi-agent chemotherapy in the first-line setting: either FOLFIRINOX administered every 2 weeks or G/N administered on days 1, 8, and 15 every 21 days or on days 1 and 8 every 15 days as tolerated. The study period was from October 2015-August 2020.

### Measures of Diabetes and Glycemic Control

Patient demographics including age, sex, race, performance status (PS), weight, body mass index (BMI), medications, and presence or absence of a diagnosis of diabetes at the time of presentation of PDAC were extracted from the hospital’s electronic medical record (EMR) PowerChart (Cerner). All random blood glucose measurements within 3 months of PDAC diagnosis were included in the baseline pre-treatment RBG (pre-RBG) and all RBG values from initiation through the first 12 months of treatment were included in the 3, 6, 9, and 12 month analyses (median RBG over the first 3 months of treatment is referred to as RBG-3 and so on). For each patient, a 3-month median RBG value was arrived at by taking the median of the three monthly average RBG values. We stratified pre-RBG and RBG-3, -6, -9, -12 values into 10 mg/dl intervals, from ≥120 through ≥180 mg/dl. Pre-treatment (baseline) HbA1c values were considered within 3 months of the start of chemotherapy and 3-month values +/- 2 weeks from that time point. Patients who survived less than 3 months were excluded from these analyses.

Patients were classified as having DM if the diagnosis or use of antidiabetic medications were listed in the medical history or in the absence of these criteria if the patient met American Association of Diabetes (ADA) criteria in the year prior based on blood glucose values present in the EMR. Diabetes diagnosed during treatment was categorized as new-onset. The duration of DM in relation to PDAC was categorized as <1 year, 1–3 years (both NOD) or >3 years (LS).

### Statistical Analysis

We focused on overall survival (OS) as the main endpoint. Overall survival was defined as the time from the start of treatment until the event (death) is reached. Patients were considered censored at the last known follow-up (August 2020) if death was not documented in the health records by that time. Kaplan-Meier and log-rank test were used to estimate OS. The circulating tumor marker CA 19-9 is elevated in approximately 80% of patients with advanced PDAC, and its reduction during treatment is a standard biomarker of response. For our analysis, a baseline elevation was considered when the value was ≥100 units/ml (≥2.7 X ULN); we also analyzed a baseline value of ≥1000 U/ml. The criteria for a 50% decline in CA 19-9 required a reduction of ≥50% in the pre-treatment value over the course of 2 consecutive months at any time during treatment with first-line chemotherapy. In addition, sex, age, body-mass index (BMI), stage, presence of liver metastases, DM status at enrollment, glucose values at the described intervals, DM type, first-line chemotherapy regimen, DM medication use, and weight loss were subjected to univariate analysis by using the log-rank test. Bivariate and multivariate Cox proportional hazard regression models were used to examine the strength of association through the estimated hazard ratios for the variables which were found significant (p<0.05) in univariate analysis and other clinically relevant variables. To reduce over- or under-estimation during multivariate analysis, only variables with no missing values for RBG-3 applicable patients were considered. To determine RBG-3 cut-off values to predict survival, receiver operating characteristic (ROC) analysis was employed to predict performance (sensitivity and specificity) of RBG-3 at each 10 mg/dl interval on 12 month survival. ROC was measured by area under the curve (AUC). All statistical analyses were performed using JMP^®^ Version 15.2 (SAS Institute Inc., Cary, NC) and SPSS^®^ Statistics Subscription Build 1.0.0.1327 (IBM, Armonk, NY).

## Results

### Patient Characteristics

Baseline patient characteristics are listed in **Table 1**. Seventy-three patients were enrolled onto our biobank study with stage III unresectable (21.9%) or stage IV (78.1%) PDAC during the time period. The median age was 72 years, 47% were female. 86.1% had an Eastern Cooperative Oncology Group performance status (ECOG PS) 0–1, 38.4% had DM and 56.9% had a BMI that was over-weight or obese. 75% of patients with stage IV disease had liver metastases. Regarding CA 19-9, 76.7% had a baseline elevation (≥100 U/ml) and 57.5% had a baseline level >1000 U/ml. The primary tumor site was categorized as being in the head (39.7%), body (42.5%) or tail (17.8%). The majority of patients received FOLFIRINOX (49.3%) or G/N (45.2%).

**Table 1:** Demographic and Clinical Characteristics of the PDAC Cohort (n=73).

Age, median (range)	72 (44–90)
Sex, n (%)	
Female	35 (47.9)
Male	38 (52.1)
ECOG PS, n (%)	
0	17 (23.2)
1	46 (63)
2	08 (11)
3	01 (1)
BMI	
median (IQR)	25.9 (7.4)
Normal (<25)	31 (43.0)
Overweight (25–29)	14 (19.4)
Obese (>29)	27 (37.5)
Diabetes, n (%)	28 (38.4)
Tumor Location, n (%)	
Head/Neck	29 (39.7)
Body	31 (42.5)
Tail	13 (17.8)
Stage, n (%)	
III	16 (21.9)
IV	57 (78.1)
Liver Metastases Stage IV, n (%)	44 (74.5)
CA 19-9	
median (range)	1390 (0–2533000)
Elevated (>100) - n (%)	56 (76.7)
Elevated (>1000) - n (%)	42 (57.5)
50% Drop at any point - n (%)	36 (49.3)
50% Drop within 3 months - n (%)	26 (35.6)
First line chemotherapy, n (%)	
FOLFIRINOX	36 (49.3)
gemcitabine/nab-paclitaxel	33 (45.2)
Others	04 (5.4)

### Survival Outcomes

**Figure 1:**
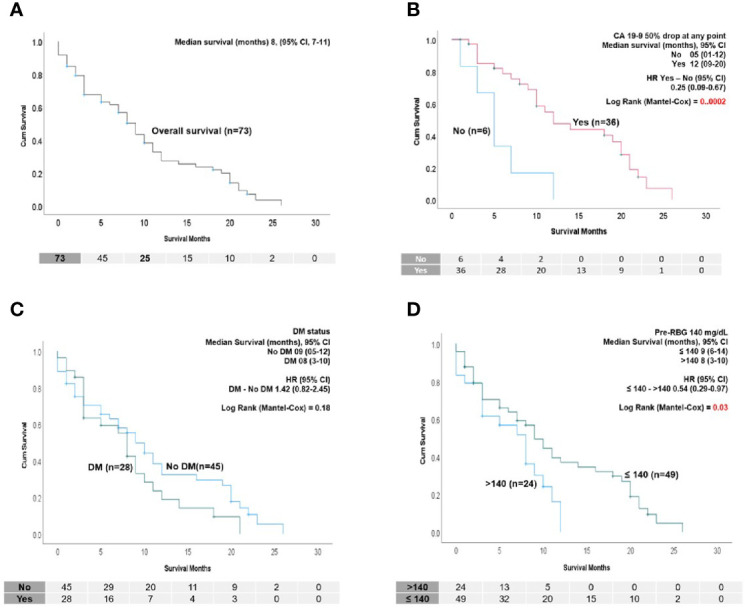
**(A)** Kaplan–Meier (K-M) survival analysis showing overall survival of the PDAC cohort in this study (n=73). **(B)** K-M survival analysis showing that patients who had 50% drop at any point after diagnosis have significantly longer survival than patients who did not. Only patients who had a baseline CA 19-9 (>100 U/ml) and survived at least 2 months were considered (n=42). **(C)** K-M survival analysis showing median survival and HR of the patients with and without DM. No survival difference was found. **(D)** Survival according to pre-RBG (≤ 140 mg/dl >). K-M survival analysis showing patients who had a pre-treatment RBG of ≤140 mg/dl survived longer than those with pre-RBG >140 mg/dl. In **(B–D)**, median survival and hazard ratio (HR) with log-rank test significance are indicated.

The median OS of the cohort was 8 months (95% CI, 7–11 months) (**Figure 1A**). Neither age, sex, BMI, stage or presence of CA 19-9 elevation at baseline were related to OS in univariate or multivariate analysis (**Table 3**). As shown in **Figure 1B**, among the patients with a baseline elevation in CA 19-9 ≥100 U/ml (and who survived at least 2 months), a 50% decline at any time during treatment was a major predictor of survival: 12 months compared to 5 months for those with and without a decline, respectively (HR=0.21, *P*=0.0002). Patients treated with the FOLFIRINOX regimen had a superior survival compared to those who received G/N; however, age was a confounding variable as 78% of those who received G/N were older than 70 years of age in comparison to only 33% of those treated with FOLFIRINOX (**Supplementary Figures 1A–C**).

### Diabetes Characteristics and Impact on Survival

**Figure 2:**
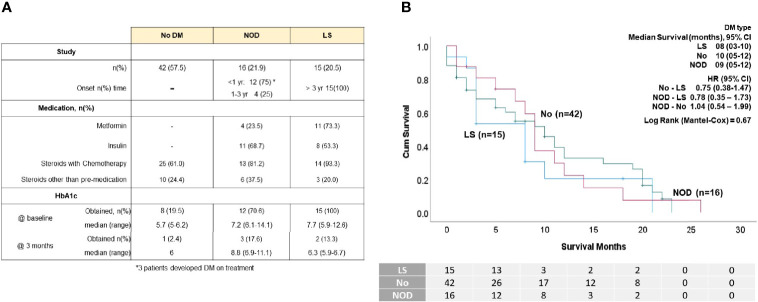
DM status of the patient cohort. **(A)** Table displaying number of patients in each group with No DM, new-onset DM (NOD) and long standing DM (LS), select medications and percentages of patients who had HbA1C levels take at baseline and at 3 months. **(B)** K-M survival analysis showing median survival and HR of the patients according to DM status. No survival differences were observed between groups.

A diagnosis of DM at the initiation of chemotherapy did not impact OS (**Figure 1C**). Twenty-eight (38.4%) patients had DM, with time of onset shown in **Figure 2A**. In those with NOD, 75% developed DM within 1 year of their PDAC diagnosis, including three patients who developed DM on treatment (two patients required hospitalization for diabetic ketoacidosis (DKA). We found no difference in survival between the NOD and LS groups (**Figure 2B**). Diabetes medication use at any time (81% at study entry) revealed that approximately 60% of those with DM were treated with insulin but metformin was more commonly utilized in the LS group (**Figure 2A**). There were no differences in OS between metformin or insulin users and non-users (data not shown). Steroid medication use was prevalent, attributable to its inclusion in standard anti-emetic regimens. Steroids were also used for other reasons, such as delayed emesis, cachexia and gemcitabine reactions (rash, pulmonary toxicity); the latter use precipitated DKA in one patient (peak glucose 897 mg/dl).

### Glycemic Control and Prognosis

We considered glycemic indices both before and during treatment for their ability to predict survival. Determination of the significance of pre-treatment RBG values in the range 120-180 mg/dl indicated that an RBG of 140 mg/dl (which corresponds to a HbA1c of 6.5%) was the most discriminatory (**Figure 1D**). Univariate analysis showed that a pre-treatment RBG ≤140 mg/dl predicted for improved OS compared to >140 mg/dl (HR=0.54, p=0.03) (**Figure 1D**), although this did not retain significance in multivariate analysis (**Table 3**). Less than half of the cohort had baseline HbA1c values performed, preventing its use as an indicator of pre-treatment glycemic control (**Figure 2A**).

To assess how glycemic control during treatment impacted OS, since less than 10% of the cohort had 3-month follow-up HbA1c levels (**Figure 2A**), we analyzed 1,410 RBG values from 3 months before through 12 months of treatment and divided these into 3-month intervals to parallel that measured by HbA1c. As shown in **Table 2**, the number of patients in each group progressively declined, leading to the most RBG values in RBG-3 (54 patients, 561 values) and the fewest in RBG-12 (18 patients, 90 values). Fifteen patients died within the first 3 months (some chose hospice after brief chemotherapy) and four patients had not yet passed the 3 month mark at the time of data cut-off (19 patients in total excluded), resulting in 54 patients eligible for RBG-3 analysis.

**Table 2:** Random Blood Glucose (RBG) values measured (total = 1418).

	pre-RBG	RBG-3	RBG-6	RBG-9	RBG=12
	3 months prior to Rx (n=73)*	0–3 months (n=54)	3–6 months (n=40)	6–9 months (n=29)	9–12 months (n=18)
**Total number of glucose measurements, n**.	282	561	289	196	90
** **					
**Average number of glucose measurements per patient, n. (range)**	3.9 (1–17)	10.4 (3–26)	7.2 (2–20) (n=39)^#^	6.7 (3–18) (n=27)^#^	5 (2–15) (n=15)^#^
** **					
**Median glucose level per pt, No-DM, (range)**	116 (82–249)	118 (82–164)	123 (98–182)	118 (83–160)	108 (87–160)
**Median glucose level per pt, NOD, (range)**	153 (114–346)	166 (97–267)	150 (91–209)	162 (89–202)	130 (84–150)
**Median glucose level per pt, LS, (range)**	164 (91–367)	182 (117–256)	185 (108–192)	129 (128–153)	133 (130–137)
	* n = number of patients, # patients with only one RBG were excluded				

**Figure 3:**
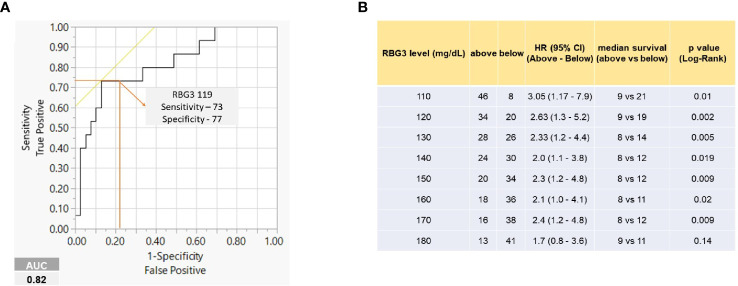
RBG-3 levels predict survival. **(A)** ROC curve showing RBG-3 of 119 with highest sensitivity (73) and specificity (77) to predict survival at 12 months (AUC 0.82). **(B)** Table shows median survival, HR and significance of RBG-3 values at 10 mg/dl intervals. 120 mg/dl shows the highest median survival differences and HR with at least 20 patients in each group.

**Figure 4:**
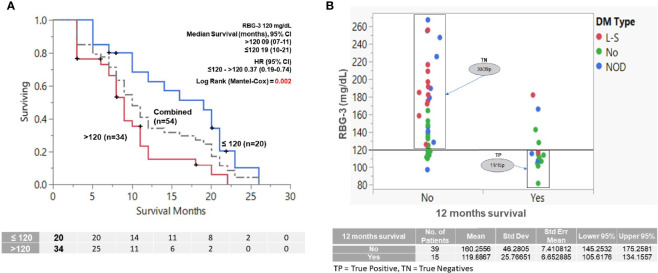
Effect of RBG-3 above or below 120 mg/dl on OS and 12 months survival (RBG-3=average glucose values of the first 3 months of treatment. N.B. Only patients who survived at least 3 months were considered for analysis (n=54). **(A)** K-M survival analysis showing that patients with RBG-3 values ≤120 mg/dl survived significantly longer (19 months) than patients with RBG-3 >120 mg/dl (9 months) (p=0.002, HR = 0.37). **(B)** One-way analysis of patients separated by RBG-3 of 120 mg/dl predicts for survival at 12 months with sensitivity of 73% (TP = 11) and specificity 77% (TN=30). Dots are color coded by DM status.

Receiver operating characteristic (ROC) analysis was implemented to determine the predictive performance of a range of RBG-3 values to predict survival at 12 months. The RBG-3 values 117 and 119 mg/dl had the highest sensitivity and specificity (AUC=0.82), leading us to choose 120 mg/dl for further analysis (**Figure 3A**). As shown in **Figure 3B**, an RBG-3 of 120 mg/dl (which corresponds to a HbA1c of 5.8%) had the most significant HR and balanced number of patients above and below this glucose level. Kaplan-Meier survival curves showed that patients with an RBG-3 ≤120 mg/dl had a superior OS of 19 months, compared to 9 months for those >120 mg/dl (**Figure 4A**) (HR=0.37, P=0.002). Twelve month survival according to DM status and RBG-3 values is shown in a one-way analysis (**Figure 4B**). Furthermore, sensitivity/specific analysis of different variables affecting survival as well as bivariate analysis of potential confounding variables confirmed the independent prognostic ability of RBG-3 120 mg/dl (**Supplementary Tables 1**, **2**). Variables in univariate analysis achieving significance at the p ≤0.05 level and other clinically relevant covariates were considered for multivariable Cox regression analysis (**Table 3**). Only RBG-3 120 mg/dl retained prognostic significance in both univariate and multivariate analyses. In contrast, a 50% decline in CA19-9 after 3 months of treatment did not predict OS.

**Table 3:** Univariate and Multivariate Analysis of Risk Factors and Impact on Overall Survival.

Characteristic	Univariate analysis				Multivariate analysis			
	No. of Patients	HR	95% CI	p value (Log-Rank)	No. of Patients	HR	95% CI	p value (Wald test)
Age (≤70 *vs.* >70)	73	0.6	0.34*–*1.06	0.08	54	0.63	0.24*–*1.63	0.63
Sex (M *vs.* F)	73	1.29	0.77*–*2.19	0.33	54	0.79	0.37*–*1.66	0.53
BMI (≤25 *vs.* >25)	72	1.13	0.67*–*1.91	0.63	54	0.78	0.35*–*1.73	0.53
Tumor stage (III *vs.* IV)	73	0.71	0.38*–*1.36	0.31	54	0.73	0.26*–*2.06	0.56
Teatment (FFRNX *vs.* G/N)	73	0.55	0.32*–*0.95	**0.03**	54	0.65	0.18*–*3.2	0.36
Liver metastases Stage IV (Yes *vs.* No)	60	1.52	0.74*–*3.16	0.26		–	–	–
Elevated CA 19-9 (>100) at study the entry (Yes *vs.* No)	73	0.84	0.45*–*1.56	0.58		–	–	–
Elevated CA 19-9 (>1000) at study the entry (Yes *vs.* No)	73	1.57	0.92*–*2.67	0.09	54	1.18	0.48*–*2.9	0.7
50% Drop in CA 19-9 at any point (Yes *vs.* No)	42	0.26	0.09*–*0.67	**0.005**		–	–	–
50% Drop in CA 19-9 @3 months after Dx (Yes *vs.* No)	37	0.67	0.32*–*1.45	0.31		–	–	–
Diabetes at the study entry (Yes *vs.* No)	73	1.42	0.83*–*2.46	0.2		–	–	–
Diabetes type								
(No *vs.* LS)	57	0.75	0.38*–*1.47	0.4	54	1.08	0.42*–*2.79	0.42
(NOD *vs.* LS)	31	0.78	0.35*–*1.73	0.55	54	1.24	0.44*–*3.51	0.67
(NOD *vs.* No)	57	1.04	0.55*–*1.99	0.89	54	1.14	0.48*–*2.7	0.75
RBG-pre avg. 3 months prior to Dx (≤140 *vs.* >140)	73	0.54	0.29*–*0.97	**0.03**	54	0.68	0.29*–*1.64	0.39
RBG avg. 3 months after Dx (≤120 *vs.* >120)	54	0.38	0.19*–*0.75	**0.005**	54	0.39	0.16*–*0.98	**0.04**
Metformin (Yes vs No)	32	1.02	0.47*–*2.22	0.94		–	–	**-**
Insulin (Yes vs No)	32	0.98	0.44*–*2.19	0.96		–	–	**-**
Other steroid use (Yes vs No)	73	0.9	0.50*–*1.61	0.72		–	–	**-**
10% drop in weight within 3 months of Dx (Yes *vs.* No)	54	1.36	0.71*–*2.64	0.35		–	–	**-**

Bold value represent statistical significance (p < 0.05).

## Discussion

In this study, we examined the relationship between glycemic control and overall survival in a cohort of patients with advanced pancreatic cancer undergoing chemotherapy treatments in a community hospital-based setting, Despite the well-studied connection between DM and PDAC, and the fact that most PDAC patients present with unresectable or metastatic disease, there is little published data on the influence of glycemic control on treatment outcomes. By analyzing over 1,400 blood glucose values and dividing them into 3-month intervals over the course of 1 year, we found that an RBG ≤120 mg/dl during the first 3 months of treatment was strongly predictive of prolonged survival (19 months *vs.* 9 for those with an RBG-3 >120 mg/d). This effect was independent of a diagnosis of DM. Furthermore, we found, as have others (25) that an early decline in CA 19-9 was not prognostic for OS, indicating that RBG-3 may be an improved early biomarker in advanced PDAC. To our knowledge, this is the first report of its kind.

We used RBG values instead of HbA1c to assess glycemic control for several reasons, including its use in a recent study (22): 1) Follow-up HbA1c values in patients with DM are often not obtained by oncologists, confirmed in this study (14); 2) Since HbA1c values are not routinely obtained from individuals without DM, reliance on HbA1c misses the impact of hyperglycemia on non-DM patients; 3) RBG values may be more accurate than HbA1c in patients with aggressive malignances such as PDAC, characterized by anemia and use of erythropoietin therapy and blood transfusions (26–28); and 4) In comparison with blood glucose levels, HbA1c is not as easily obtainable in resource limited settings (29).

The results of this study extend accumulating evidence linking abnormal glycemic indices and/or a diagnosis of DM to poor PDAC outcomes. In the resectable disease setting, a pre-treatment HbA1c >6.5% was associated with failure to complete neoadjuvant chemotherapy and surgery (21) and hyperglycemia (glucose >200 mg/dl) was associated with reduced OS in patients with non-metastatic PDAC undergoing stereotactic body radiation therapy (22). Diabetes mellitus and in particular NOD predicted for significantly reduced survivals following surgical resection in recent series (17, 30) and a meta-analysis (31) although this finding is not universal (32). In the metastatic disease setting, DM (NOD or LS), an initial fasting blood glucose ≥126 mg/dl and HbA1c >7.0% have all been found to be independent predictors for increased risk of death (18, 33–35). We found that OS was negatively impacted by an elevated pre-treatment RBG ≥140 mg/dl (significant in univariate analysis), which is consistent with the above reports.

According to the ADA, an RBG <140 mg/dl is normal, 140-200 mg/dl is pre-diabetic and >200 mg/dl meets criteria for DM (36). Our finding that RBG levels in the normal range during the first 3 months of chemotherapy are associated with prolonged survivals for advanced PDAC patients (median 19 months) has not been previously reported and was unexpected. It may also not seem relevant, as modest elevations of random glucose values are commonly discounted by busy clinicians in real-world practice. Yet, a study of 13,792 participants in the National Health and Nutrition Examination Surveys (NHANES) found that a single RBG ≥100 mg/dl conferred a significant risk for undiagnosed DM (37). There are also reports that elevated glucose and HbA1c levels within the normal range are associated with increased cancer risk and mortality (38–40). In a recent study of 572,021 Korean adults without cancer at baseline, glycemic status in the nondiabetic range, insulin resistance and hyperinsulinemia were each independently associated with increased mortality from PDAC (38). In nondiabetic women with locally advanced cervical cancer, a pre-treatment RBG >102 mg/dl predicted for worse outcomes (39). These observations support the physiological relevance of even modest elevations in RBG values, such as we report herein, and suggest that larger studies be performed to further investigate a possible link between normal range RBG-3 values and improved prognosis in advanced PDAC.

The preference of PDAC cells for energy generation through the glycolytic pathway renders an ample supply of glucose essential for optimal growth and survival, as previously discussed. Indeed, there exists an abundance of scientific evidence regarding the ill-effects of hyperglycemia on cancer growth and clinical outcomes [reviewed in (10, 12, 15, 41, 42)]. In addition, hyperglycemia leads to increased blood levels of insulin and insulin-like growth factors (IGF), which promote tumor growth and are targets of novel therapeutics (43).

On the other hand, the potential beneficial effects of low-normal glucose levels has been far less investigated although there is preliminary *in-vitro* evidence (44, 45). Whether efforts aimed at preventing hyperglycemia, either through dietary changes, such as the ketogenic diet (46) or medications, such as metformin, would favorably impact PDAC outcomes remains unclear; one study of metformin plus chemotherapy did not find a benefit (47). Based on our findings, however, we do recommend consideration of eliminating the commonly utilized steroid medication dexamethasone from chemotherapy anti-emetic regimens for some PDAC patients owing to their hyperglycemic effects (48).

Whether good glycemic control (RBG of 120 mg/dl corresponds to a HbA1c of 5.8%) facilitates tumor responsiveness to chemotherapy or responding tumors favorably impact glucose metabolism could not be answered by this study. Since resection of localized PDAC in individuals with NOD often leads to resolution of the DM (20), our findings would support the notion that in the metastatic setting, improved tumor control leads to improved glycemic control.

The strengths of our study include close monitoring of patients in a community setting and a cohort more reflective of an unselected population than is seen at larger, tertiary cancer centers. The main limitation of our study is the relatively small sample size, which we attribute to two main factors: 1) Patients were drawn from two mid-sized community hospital cancer centers rather than from larger centers; and 2) Our cohort did not include all PDAC patients treated at our hospitals. In order to evaluate as homogeneous a patient cohort as possible, we restricted our analysis to patients with advanced PDAC able to undergo multi-agent chemotherapy and who agreed to participate in a biobanking clinical trial. Therefore, patients with resectable or borderline resectable disease able to undergo resection after neoadjuvant chemotherapy, or those unable or unwilling to be treated with multi-agent chemotherapy were not included. Despite this, our patient cohort is typical of those found in larger series, including the percentage of patients with DM (38%), percentage with liver metastases (75% of stage IV patients) and that OS was highly correlated with a 50% decline in the tumor marker CA 19-9 at any time during treatment (23–25). A second limitation is the fact that 20% of our patients died within the first 3 months. This latter observation may also be more typical of PDAC in the “real-world” although it is not captured in first-line treatment trials, which enroll only the most robust patients. A third limitation is the fact that we could not control for the varying number of RBG samples per patient nor the time of day at which the samples were obtained. We did observe that more glucose levels were obtained from those patients with hyperglycemia, owing to the need to monitor such patients more closely; this likely improved the accuracy of the average monthly RBG values for these patients.

In conclusion, the current study demonstrates that good glycemic control during the first 3 months of chemotherapy treatment for advanced PDAC predicts for improved outcomes. If confirmed in a larger series, RBG-3 would be a novel, early prognostic factor, adaptable in resource limited settings. These results add to accumulating evidence that glycemic indices, in addition to the duration and prevalence of DM, be considered as prognostic factors in future treatment trials. Further study, most notably exploring possible cause and effect of this novel survival-linked relationship, seems warranted.

## Data Availability Statement

The raw data supporting the conclusions of this article will be made available by the authors, without undue reservation.

## Ethics Statement

The studies involving human participants were reviewed and approved by BRANY Institutional Review Board: www.brany.com N.B. The reported study data are the result of a record review of patients enrolled in a biobanking clinical trial. This trial is registered on clinicaltrials.gov (ID in manuscript) and approved by the above IRB, but the results reported in this paper were not specified as part of this clinical trial. Consent to obtain demographic and laboratory data were part of the trial informed consent. The patients/participants provided their written informed consent to participate in this study.

## Author Contributions

IA, RS, BB, DP, and RF contributed to conception and design of the study. IA, RS, MD, SE, and TL performed data acquisition and organized the database. DP performed the statistical analysis. RF wrote the first draft of the manuscript. IA, BB, DP, and RF wrote sections of the manuscript. All authors contributed to the article and approved the submitted version.

## Funding

This work was supported by the Ron Foley Foundation, the Rallye for Pancreatic Cancer and a Tribute to Pamela/The Naughton Family.

## Conflict of Interest

The authors declare that the research was conducted in the absence of any commercial or financial relationships that could be construed as a potential conflict of interest.

## Supplementary Material

Supplementary Figure 1 **(A)** Survival according to baseline treatment. K-M survival analysis showing patients who were treated with the FOLFIRINOX regimen had a longer survival compared to those who received G/N. (11 *vs.* 6 months, p=0.03, HR = 1.78). **(B)** 2 x 2 table showing highly significant age difference by treatment regimen. **(C)** Bivariate analysis demonstrating the confounding effect of age on OS by treatment regimen.
