# Supplementary figures and images for: Glycemic Control as an Early Prognostic Marker in Advanced Pancreatic Cancer
Source: Front Oncol. 2021 Feb 25;11:571855. doi: 10.3389/fonc.2021.571855 (PMC7947820; doi:10.3389/fonc.2021.571855)

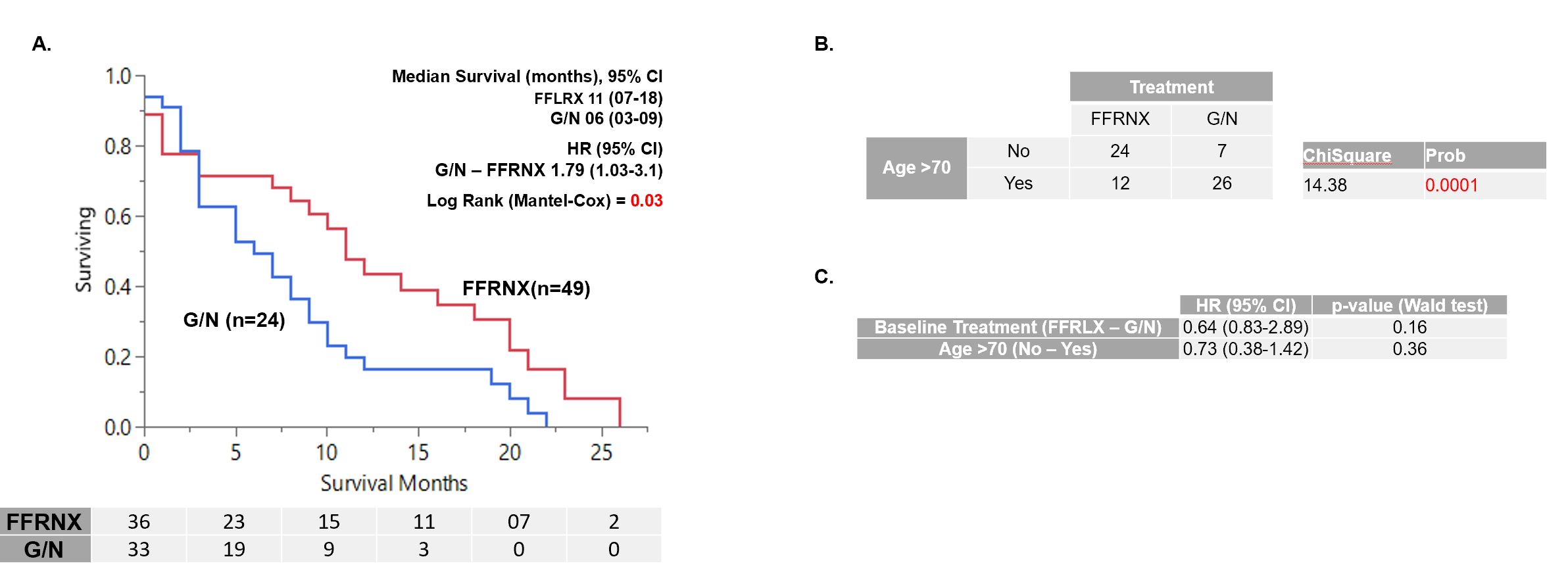

Supplement: Supplementary Figure 1 — (A) Survival according to baseline treatment. K-M survival analysis showing patients who were treated with the FOLFIRINOX regimen had a longer survival compared to those who received G/N. (11 vs. 6 months, p=0.03, HR = 1.78). (B) 2 x 2 table showing highly significant age difference by treatment regimen. (C) Bivariate analysis demonstrating the confounding effect of age on OS by treatment regimen. [file Image_1.jpg]
